# 2,4-Bis(4-fluoro­phen­yl)-3-aza­bicyclo­[3.3.1]nonan-9-one

**DOI:** 10.1107/S1600536808024744

**Published:** 2008-08-06

**Authors:** P. Parthiban, V. Ramkumar, H. D. Santan, Jong Tae Kim, Yeon Tae Jeong

**Affiliations:** a Division of Image Science and Information Engineering, Pukyong National University, Busan 608 739, Republic of Korea; b Department of Chemistry, IIT Madras, Chennai, Tamilnadu, India

## Abstract

In the title compound, C_20_H_19_F_2_NO, a crystallographic mirror plane bis­ects the mol­ecule, passing through the N, O and two C atoms of the central ring system. The mol­ecule exists in a twin-chair conformation with equatorial dispositions of the 4-fluoro­phenyl groups on both sides of the secondary amino groups; the dihedral angle between the aromatic ring planes is 28.67 (3)°.

## Related literature

For chemical background, see: Buxton & Roberts (1996 ▶); Evans & Seddon (1997 ▶); Ramachandran *et al.* (2007 ▶). For ring puckering parameters, see: Cremer & Pople (1975 ▶).
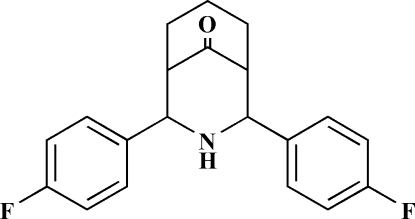


## Experimental

### 

#### Crystal data

C_20_H_19_F_2_NO
*M* *_r_* = 327.36
Orthorhombic, 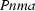

*a* = 7.6153 (3) Å
*b* = 21.1392 (9) Å
*c* = 10.0878 (4) Å
*V* = 1623.95 (11) Å^3^
*Z* = 4
Mo *K*α radiation
μ = 0.10 mm^−1^
*T* = 298 (2) K
0.35 × 0.32 × 0.30 mm

#### Data collection

Bruker APEXII CCD diffractometer
Absorption correction: multi-scan (*SADABS*; Bruker, 2004 ▶) *T*_min_ = 0.967, *T*_max_ = 0.971
11360 measured reflections
2064 independent reflections
1596 reflections with *I* > 2σ(*I*)
*R*_int_ = 0.020

#### Refinement

*R*[*F* ^2^ > 2σ(*F* ^2^)] = 0.041
*wR*(*F* ^2^) = 0.141
*S* = 0.91
2064 reflections
118 parameters
H atoms treated by a mixture of independent and constrained refinement
Δρ_max_ = 0.21 e Å^−3^
Δρ_min_ = −0.19 e Å^−3^

### 

Data collection: *APEX2* (Bruker, 2004 ▶); cell refinement: *SAINT* (Bruker, 2004 ▶); data reduction: *SAINT*; program(s) used to solve structure: *SHELXS97* (Sheldrick, 2008 ▶); program(s) used to refine structure: *SHELXL97* (Sheldrick, 2008 ▶); molecular graphics: *ORTEP-3* (Farrugia, 1997 ▶); software used to prepare material for publication: *SHELXL97*..

## Supplementary Material

Crystal structure: contains datablocks global, I. DOI: 10.1107/S1600536808024744/hb2763sup1.cif

Structure factors: contains datablocks I. DOI: 10.1107/S1600536808024744/hb2763Isup2.hkl

Additional supplementary materials:  crystallographic information; 3D view; checkCIF report

## Figures and Tables

**Fig. 1.**
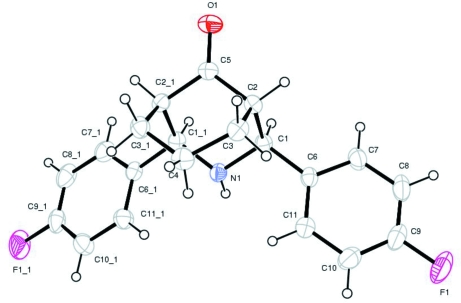
The molecular structure of (I) with non-hydrogen atoms represented as 30% probability ellipsoids.

## supplementary crystallographic information

### Comment

Fluorine substituted organic compounds are very impotant due to the significance of C—F bonds in some bioorganic systems (e.g. Evans & Seddon, 1997). The intermolecular and intramolecular hydrogen bonds involving fluorine atom have attracted much attention in various aspects (e.g. Ramachandran *et al.*, 2007). Moreover, the biological activities mainly depend on the stereochemistry of the synthesized compound (e.g. Buxton & Roberts, 1996). Hence, realising the importance of the investigation of the conformation, stereochemistry and the nature of bondings in the synthesized title fluorine substituted heterocycle, (I), we have carried out single-crystal X-ray diffraction studies.

An analysis of torsion angles, asymmetry parameters and least-squares plane calculation shows that the piperidine ring adopts a near ideal chair conformation with the deviation of ring atoms N1 and C5 from the C1/C1^i^/C2/C2^i^ (i = x, 1/2-y, z) plane by -0.670 (3)Å and 0.693 (3)Å respectively, Q_T_ = 0.6064 (13) Å. The cyclohexane ring deviate from the ideal chair conformation by the deviation of ring atoms C4 and C5 from the C2/C2^i^/C3/C3^i^ plane by 0.522 (4)Å and 0.734 (3)Å respectively, Q_T_ = 0.5681 (14)Å (Cremer & Pople, 1975).

Compound (I) has a crystallographic mirror plane, which bisects the molecule passing through N1, C4, C5 and O1 of the central ring (Fig. 1) and exists in twin-chair conformation with equatorial orientations of the *para* fluoro phenyl groups on the heterocycle with the torsion angle of C5—C2—C1—C6 is 178.41 (6)°. The aryl groups are orientated at an angle of 28.67 (3)° to each other.

### Experimental

A mixture of cyclohexanone (0.05 mol) and *para* fluorobenzaldehyde (0.1 mol) was added to a warm solution of ammonium acetate (0.075 mol) in 50 ml of absolute ethanol. The mixture was very gently warmed on a hot plate till the yellow color formed during the mixing of the reactants and allowed to stir till the formation of the product. Thus, the formed azabicyclononane was separated by filtration and washed with a 1:5 v/v ethanol-ether mixture till the solid became colorless. Then, recrystallization of the compound from ethanol afforded colorless blocks of (I).

### Refinement

The nitrogen H atom was located in a difference Fourier map and refined isotropically. Other hydrogen atoms were fixed geometrically (C—H = 0.93-0.97Å) and refined as riding with *U*_iso_(H) = 1.2*U*_eq_(C).

### Crystal data

**Table:**

C_20_H_19_F_2_NO	*F*(000) = 688
*M**_r_* = 327.36	*D*_x_ = 1.339 Mg m^−^^3^
Orthorhombic, *P**n**m**a*	Mo *K*α radiation, λ = 0.71073 Å
Hall symbol: -P 2ac 2n	Cell parameters from 4451 reflections
*a* = 7.6153 (3) Å	θ = 3.4–28.0°
*b* = 21.1392 (9) Å	µ = 0.10 mm^−^^1^
*c* = 10.0878 (4) Å	*T* = 298 K
*V* = 1623.95 (11) Å^3^	Block, colourless
*Z* = 4	0.35 × 0.32 × 0.30 mm

### Data collection

**Table:**

Bruker APEXII CCD diffractometer	2064 independent reflections
Radiation source: fine-focus sealed tube	1596 reflections with *I* > 2σ(*I*)
graphite	*R*_int_ = 0.020
ω scans	θ_max_ = 28.3°, θ_min_ = 1.9°
Absorption correction: multi-scan (*SADABS*; Bruker, 2004)	*h* = −8→10
*T*_min_ = 0.967, *T*_max_ = 0.971	*k* = −28→28
11360 measured reflections	*l* = −13→12

### Refinement

**Table:**

Refinement on *F*^2^	Primary atom site location: structure-invariant direct methods
Least-squares matrix: full	Secondary atom site location: difference Fourier map
*R*[*F*^2^ > 2σ(*F*^2^)] = 0.041	Hydrogen site location: inferred from neighbouring sites
*wR*(*F*^2^) = 0.141	H atoms treated by a mixture of independent and constrained refinement
*S* = 0.91	*w* = 1/[σ^2^(*F*_o_^2^) + (0.0897*P*)^2^ + 0.3733*P*] where *P* = (*F*_o_^2^ + 2*F*_c_^2^)/3
2064 reflections	(Δ/σ)_max_ < 0.001
118 parameters	Δρ_max_ = 0.22 e Å^−^^3^
0 restraints	Δρ_min_ = −0.19 e Å^−^^3^

### Special details

**Table:**

Geometry. All e.s.d.'s (except the e.s.d. in the dihedral angle between two l.s. planes) are estimated using the full covariance matrix. The cell e.s.d.'s are taken into account individually in the estimation of e.s.d.'s in distances, angles and torsion angles; correlations between e.s.d.'s in cell parameters are only used when they are defined by crystal symmetry. An approximate (isotropic) treatment of cell e.s.d.'s is used for estimating e.s.d.'s involving l.s. planes.
Refinement. Refinement of *F*^2^ against ALL reflections. The weighted *R*-factor *wR* and goodness of fit *S* are based on *F*^2^, conventional *R*-factors *R* are based on *F*, with *F* set to zero for negative *F*^2^. The threshold expression of *F*^2^ > σ(*F*^2^) is used only for calculating *R*-factors(gt) *etc*. and is not relevant to the choice of reflections for refinement. *R*-factors based on *F*^2^ are statistically about twice as large as those based on *F*, and *R*- factors based on ALL data will be even larger.

### Fractional atomic coordinates and isotropic or equivalent isotropic displacement parameters (Å^2^)

**Table:**

	*x*	*y*	*z*	*U*_iso_*/*U*_eq_	
C1	0.92315 (17)	0.30735 (5)	0.39201 (12)	0.0391 (3)	
H1	0.8839	0.3055	0.2996	0.047*	
C2	0.75726 (16)	0.30901 (5)	0.48153 (14)	0.0421 (3)	
H2	0.6864	0.3460	0.4576	0.051*	
C3	0.79266 (19)	0.31054 (6)	0.63140 (13)	0.0473 (3)	
H3A	0.8701	0.3458	0.6508	0.057*	
H3B	0.6827	0.3181	0.6773	0.057*	
C4	0.8751 (3)	0.2500	0.68562 (18)	0.0492 (4)	
H4A	0.9993	0.2500	0.6644	0.059*	
H4B	0.8642	0.2500	0.7814	0.059*	
C5	0.6527 (2)	0.2500	0.45348 (18)	0.0435 (4)	
C6	1.03437 (17)	0.36590 (5)	0.40791 (12)	0.0400 (3)	
C7	0.9916 (2)	0.41937 (6)	0.33533 (16)	0.0543 (4)	
H7	0.8976	0.4179	0.2766	0.065*	
C8	1.0856 (2)	0.47481 (7)	0.34848 (19)	0.0666 (5)	
H8	1.0550	0.5107	0.3003	0.080*	
C9	1.2238 (2)	0.47573 (7)	0.4334 (2)	0.0643 (5)	
C10	1.2733 (2)	0.42414 (8)	0.50616 (19)	0.0641 (4)	
H10	1.3688	0.4261	0.5634	0.077*	
C11	1.1771 (2)	0.36882 (7)	0.49220 (15)	0.0519 (4)	
H11	1.2091	0.3331	0.5404	0.062*	
F1	1.31755 (17)	0.53023 (5)	0.44594 (17)	0.1030 (5)	
H111	1.121 (3)	0.2500	0.369 (2)	0.047 (5)*	
N1	1.0241 (2)	0.2500	0.41923 (15)	0.0384 (3)	
O1	0.50237 (19)	0.2500	0.41536 (17)	0.0626 (4)	

### Atomic displacement parameters (Å^2^)

**Table:**

	*U*^11^	*U*^22^	*U*^33^	*U*^12^	*U*^13^	*U*^23^
C1	0.0441 (7)	0.0372 (6)	0.0359 (6)	0.0013 (5)	−0.0009 (5)	0.0021 (4)
C2	0.0395 (6)	0.0368 (6)	0.0500 (7)	0.0045 (5)	−0.0010 (5)	0.0018 (5)
C3	0.0493 (7)	0.0465 (7)	0.0461 (7)	−0.0017 (6)	0.0077 (6)	−0.0072 (5)
C4	0.0529 (10)	0.0585 (10)	0.0361 (9)	0.000	0.0003 (8)	0.000
C5	0.0384 (9)	0.0483 (9)	0.0437 (9)	0.000	−0.0027 (7)	0.000
C6	0.0440 (7)	0.0352 (5)	0.0406 (6)	0.0016 (5)	0.0073 (5)	0.0021 (4)
C7	0.0567 (8)	0.0455 (7)	0.0605 (8)	0.0048 (6)	0.0044 (7)	0.0137 (6)
C8	0.0694 (10)	0.0390 (7)	0.0915 (12)	0.0051 (7)	0.0242 (10)	0.0157 (7)
C9	0.0586 (9)	0.0382 (7)	0.0961 (13)	−0.0091 (6)	0.0292 (9)	−0.0104 (7)
C10	0.0558 (9)	0.0579 (9)	0.0787 (11)	−0.0105 (7)	0.0011 (8)	−0.0110 (8)
C11	0.0529 (8)	0.0455 (7)	0.0572 (8)	−0.0038 (6)	−0.0029 (6)	0.0043 (6)
F1	0.0834 (8)	0.0474 (6)	0.1782 (14)	−0.0225 (5)	0.0323 (8)	−0.0170 (6)
N1	0.0377 (8)	0.0341 (7)	0.0434 (8)	0.000	0.0057 (6)	0.000
O1	0.0421 (8)	0.0693 (10)	0.0763 (11)	0.000	−0.0159 (7)	0.000

### Geometric parameters (Å, °)

**Table:**

C1—N1	1.4613 (14)	C5—C2^i^	1.5067 (15)
C1—C6	1.5083 (16)	C6—C11	1.381 (2)
C1—C2	1.5533 (18)	C6—C7	1.3856 (17)
C1—H1	0.9800	C7—C8	1.380 (2)
C2—C5	1.5067 (15)	C7—H7	0.9300
C2—C3	1.536 (2)	C8—C9	1.357 (3)
C2—H2	0.9800	C8—H8	0.9300
C3—C4	1.5266 (17)	C9—F1	1.3613 (17)
C3—H3A	0.9700	C9—C10	1.367 (3)
C3—H3B	0.9700	C10—C11	1.387 (2)
C4—C3^i^	1.5266 (17)	C10—H10	0.9300
C4—H4A	0.9700	C11—H11	0.9300
C4—H4B	0.9700	N1—C1^i^	1.4613 (14)
C5—O1	1.208 (2)	N1—H111	0.89 (2)
			
N1—C1—C6	111.44 (10)	O1—C5—C2	124.12 (7)
N1—C1—C2	109.70 (10)	O1—C5—C2^i^	124.12 (7)
C6—C1—C2	112.11 (9)	C2—C5—C2^i^	111.76 (14)
N1—C1—H1	107.8	C11—C6—C7	118.28 (12)
C6—C1—H1	107.8	C11—C6—C1	122.96 (11)
C2—C1—H1	107.8	C7—C6—C1	118.76 (12)
C5—C2—C3	107.16 (11)	C8—C7—C6	121.34 (15)
C5—C2—C1	107.58 (11)	C8—C7—H7	119.3
C3—C2—C1	115.47 (11)	C6—C7—H7	119.3
C5—C2—H2	108.8	C9—C8—C7	118.37 (14)
C3—C2—H2	108.8	C9—C8—H8	120.8
C1—C2—H2	108.8	C7—C8—H8	120.8
C4—C3—C2	114.02 (11)	C8—C9—F1	118.51 (16)
C4—C3—H3A	108.7	C8—C9—C10	122.75 (14)
C2—C3—H3A	108.7	F1—C9—C10	118.74 (18)
C4—C3—H3B	108.7	C9—C10—C11	118.19 (16)
C2—C3—H3B	108.7	C9—C10—H10	120.9
H3A—C3—H3B	107.6	C11—C10—H10	120.9
C3^i^—C4—C3	113.91 (16)	C6—C11—C10	121.05 (14)
C3^i^—C4—H4A	108.8	C6—C11—H11	119.5
C3—C4—H4A	108.8	C10—C11—H11	119.5
C3^i^—C4—H4B	108.8	C1^i^—N1—C1	112.11 (14)
C3—C4—H4B	108.8	C1^i^—N1—H111	109.1 (7)
H4A—C4—H4B	107.7	C1—N1—H111	109.1 (7)
			
N1—C1—C2—C5	−58.02 (14)	C2—C1—C6—C7	−84.10 (15)
C6—C1—C2—C5	177.61 (10)	C11—C6—C7—C8	−1.5 (2)
N1—C1—C2—C3	61.57 (13)	C1—C6—C7—C8	178.21 (13)
C6—C1—C2—C3	−62.80 (13)	C6—C7—C8—C9	0.9 (2)
C5—C2—C3—C4	52.64 (16)	C7—C8—C9—F1	179.55 (15)
C1—C2—C3—C4	−67.17 (15)	C7—C8—C9—C10	0.0 (3)
C2—C3—C4—C3^i^	−43.3 (2)	C8—C9—C10—C11	−0.2 (3)
C3—C2—C5—O1	113.4 (2)	F1—C9—C10—C11	−179.77 (15)
C1—C2—C5—O1	−121.84 (19)	C7—C6—C11—C10	1.3 (2)
C3—C2—C5—C2^i^	−65.39 (17)	C1—C6—C11—C10	−178.44 (14)
C1—C2—C5—C2^i^	59.35 (18)	C9—C10—C11—C6	−0.4 (2)
N1—C1—C6—C11	−27.77 (17)	C6—C1—N1—C1^i^	−174.53 (8)
C2—C1—C6—C11	95.62 (15)	C2—C1—N1—C1^i^	60.72 (16)
N1—C1—C6—C7	152.51 (13)		

Symmetry codes: (i) *x*, −*y*+1/2, *z*.
